# What makes space-time interactions in human vision asymmetrical?

**DOI:** 10.3389/fpsyg.2015.00756

**Published:** 2015-06-08

**Authors:** Chizuru T. Homma, Hiroshi Ashida

**Affiliations:** Graduate School of Letters, Kyoto UniversityKyoto, Japan

**Keywords:** space-time interaction, temporal cognition, spatial cognition, saliency, human vision, task difficulty

## Abstract

The interaction of space and time affects perception of extents: (1) the longer the exposure duration, the longer the line length is perceived and vice versa; (2) the shorter the line length is, the shorter the exposure duration is perceived. Previous studies have shown that space-time interactions in human vision are asymmetrical; spatial cognition has a larger effect on temporal cognition rather than vice versa (Merritt et al., 2010). What makes the interactions asymmetrical? In this study, participants were asked to judge exposure duration of lines that differed in length or to judge the lengths of the lines with different exposure time; to judge the task-relevant stimulus extents that also varied in the task-irrelevant stimulus extents. Paired spatial and temporal tasks in which the ranges of task-relevant and -irrelevant stimulus values were common, were conducted. In our hypothesis, the imbalance in saliency of spatial and temporal information would cause asymmetrical space-time interaction. To assess the saliency, task difficulty was rated. If saliency of relevant stimuli is high, the difficulty of discrimination task would be low, and vice versa. The saliency of irrelevant stimuli in one task would be reflected in the difficulty of the other task, in the pair of tasks. If saliency of irrelevant stimuli is high, the difficulty of paired task would be low, and vice versa. The result supports our hypothesis; spatial cognition asymmetrically affected on temporal cognition when the difficulty of temporal task was significantly higher than that of spatial task.

## Introduction

When people imagine that they are spending their time in a small room model, like a doll's house, they tends to feel the time shorter in a smaller room model compared to the estimated time in a larger room model (DeLong, 1981; Mitchell and Davis, 1987). Spatial extents of room model can alter subjective time. It is also known that more time was required to scan across mental images with greater distances, and to scan subjectively larger images (Mental Scanning; Kosslyn, 1973; Kosslyn et al., 1978). These are examples that show interactions between spatial and temporal cognition.

There are also cognitive interactions between number and space dimensions. In a numerosity discrimination task to compare two numbers, participants can react more rapidly when numerical and spatial extents are congruent, high digit with large size and low digit with small size, than when they are incongruent, high digit with small size and low digit with large size (Henik and Tzelgov, 1982). Many other cognitive interactions, like above, among three different dimensions (space, time and number), has been reported (e.g., Vicario, 2011; Javadi and Aichelburg, 2012). Accordingly, common mechanisms to process magnitude information of space, time and number has been suggested (a theory of magnitude; ATOM, Walsh, 2003).

The research topic of this study is on the cognitive interactions between space and time dimensions: (1) the longer the exposure duration is, the longer the line length tends to be judged and vice versa; (2) the shorter the line length is, the shorter the exposure duration tends to be judged and vice versa. Previous studies have repeatedly shown asymmetrical space-time interactions in vision of human adults; spatial cognition has a larger effect on temporal cognition rather than vice versa (Casasanto and Boroditsky, 2008; Merritt et al., 2010). However, such interactions in monkeys have been shown to be symmetrical (Merritt et al., 2010). In addition, space-time interactions in vision of human infants might be symmetrical. In 9-month-age infants, learning could be transferred among the three dimensions of time, space and number in vision, equally in each direction (Lourenco and Longo, 2010): learning of an arbitrary combination in one dimension, such as a stripe pattern of visual stimuli associated with a short exposure duration, can be transferred to the other dimension in every direction to a similar extent. Then, what is the difference between human adults and monkeys? How does the balance between space-time interactions in vision of human adults differ from that of infants? Trying to answer these questions is important for better understandings of spatial and temporal cognitions, the cognitive interaction and the development. The present study approaches to the specific question; what makes the interactions asymmetrical in vision of human adults?

As mentioned above, previous works have shown asymmetrical space-time interactions in vision of human adults; spatial cognition affects time cognition more than vice versa in discrimination tasks (Casasanto and Boroditsky, 2008; Merritt et al., 2010). One problem is that the balance of spatial and temporal information in the experimental stimuli has not been considered much. Therefore, in vision of human adults, the saliencies of spatial and temporal information might be one of the factors that make space-time interactions asymmetrical.

Many studies on cross-modal audiovisual interaction have shown the predominance of vision over audition (e.g., Thurlow and Jack, 1973; Kitagawa and Ichihara, 2002). However, when the ambiguity of visual information is high and the saliency of auditory information is high, auditory information could affect visual perception was presented later (Shimojo et al., 2001; Vroomen et al., 2004). A similar phenomenon has been found in the space-time cognitions, the saliency of stimuli should affect the balance between the space-time interactions.

Cai and Connell (2015) showed that time cognition can asymmetrically affect space cognition: spatial information from haptic perception can be affected by temporal information from audition but not vice versa. However, when spatial information from vision was added, space-time cognitions affect each other to a similar extent. According to them, the balance of space-time interaction could be affected by the perceptual acuity of the modality to perceive spatial information. The results indicated that the saliency of stimuli could affect the balance of space-time interaction in multi-modal perception.

**Figure 1:**
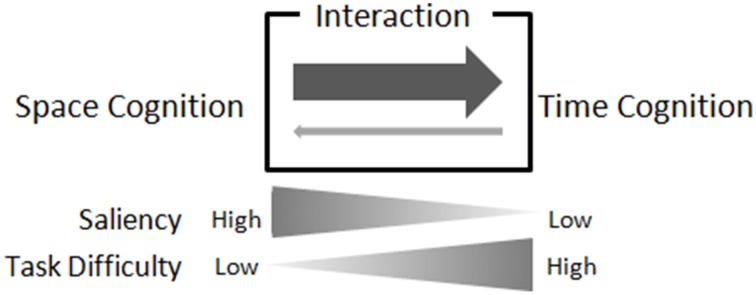
**Balance of saliency, task difficulty, and spatial/temporal interaction**. In our hypothesis, asymmetrical space-time interaction is caused by the imbalance in saliency of the spatial and temporal information, and the imbalance in difficulties in the spatial and temporal tasks. When the saliency of relevant stimuli is high, the automaticity in cognitive processes, such as discrimination, could be high, and thus the task difficulty could be low; and vice versa. The saliency of irrelevant stimuli in one task would be reflected in the difficulty of the other task, in the pair of spatial/temporal tasks. If saliency of irrelevant stimuli is high, the difficulty of paired task would be low, and vice versa. When space cognition asymmetrically affects time cognition, saliency of spatial information would be high and/or saliency of temporal information would be low.

Thus, the present study aimed to investigate the effects of saliencies of spatial and temporal information in space-time interactions in vision. We conducted an experiment in which the tasks to discriminate exposure durations or line lengths. In order to assess space-time interactions, we adapted the method of Merritt et al. (2010); line lengths were varied during duration judgments, and durations for presenting line stimuli were also varied during judgments of line lengths, and the method of Droit-Volet and Zélanti (2013); anchor stimuli, longest and shortest stimuli, were presented several times before an anchor training section. Task difficulty was rated to assess saliencies of spatial and temporal information. In a simple discrimination task, when the saliency of relevant stimuli is low, the automaticity in cognitive processes, such as discrimination, could be low, and thus the task difficulty could be high. Our hypothesis was that asymmetrical space-time interaction is caused by the imbalance in saliency of the spatial and temporal information, and the imbalance in difficulties in the spatial and temporal tasks (see Figure 1). In order to see the effect of saliency on the extent of interaction, two sets of paired spatial and temporal tasks that would be differed in the balance of difficulty (the pair that one task was more difficult than the other task, and the pair that two tasks were both difficult to the similar extent) were conducted. In each pair, the ranges of stimulus values were same; the shortest and the longest relevant stimuli in one task were same extents as the shortest and the longest irrelevant stimuli in the other task, in a pair. Thus, the difficulty of paired task would indicate the saliency of the irrelevant stimuli.

## Methods

### Participants

Twenty four adults (12 males, mean age: 23.13 years, *SD* = 3.13) performed four tasks; two line length judgment tasks and two duration judgment tasks. All the participants had normal or corrected-to-normal vision. They were paid for the time by the standard of Kyoto University. The experiments were conducted in conformity to the standards of ethical review committee in Kyoto University. Through ethical considerations, before the experiments, the content of the experiment and the rights of participants were explained, and the participants were asked to sign with agreement documents if they understood and agreed to participate in the experiment.

### Stimuli

**Figure 2:**
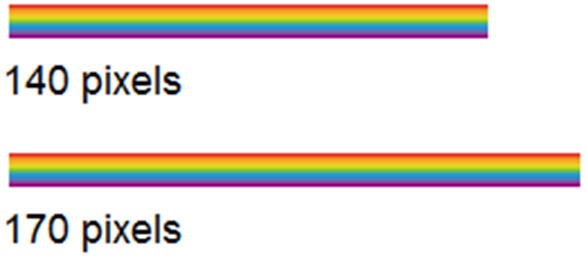
**Sample line stimuli**. The upper line was a short anchor stimuli (width: 140 pixels) and the downer line was a long anchor stimuli (width: 170 pixels) in line length judgment tasks. These were also used in duration judgment tasks. The heights of all line stimuli were 9 pixels.

**Figure 3:**
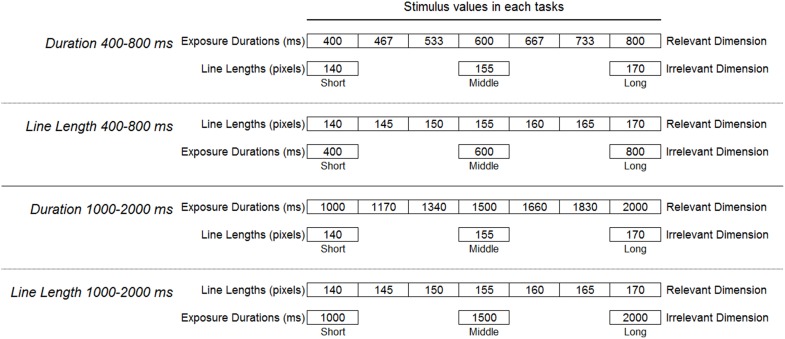
**Exposure durations and widths of line stimuli**. In anchor training and bisection testing, the values of irrelevant dimension were fixed at middle levels. In cross-dimensional testing, the values of irrelevant dimension were varied in three levels; short, middle, and long.

Rainbow colored line stimuli were presented against a gray background (Figure 2). Similar experiments were planned for children, and the stimuli were colored in order to attract their attentions. The width of line stimuli was varied within the range of 140–170 pixels, and the exposure duration was varied within the range of 400–800 ms, or 1000–2000 ms. To make the balance of task difficulties different in two pairs of duration and line length judgment tasks, the exposure duration was varied within the above two ranges. In the line length judgment tasks, seven different widths of line stimuli were presented for three different durations. In the duration judgment task, three different widths of line stimuli were presented for seven different durations. The stimulus extents of relevant dimension had seven levels and that of irrelevant dimension had three levels. In each task, there were 21 stimulus presentation patterns (Figure 3). The line stimuli were presented on a 13-inch LCD display with a resolution of 1024 × 768 pixels.

### Procedure

**Figure 4:**
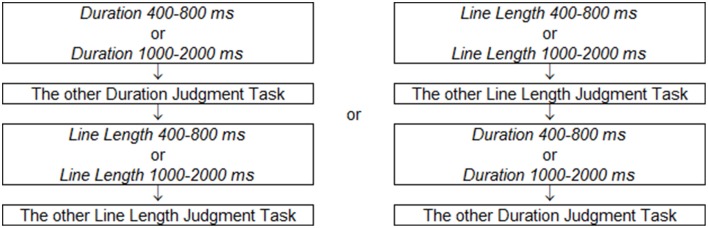
**The order of task blocks**. Half of participants finished two line length judgment tasks ahead and two duration judgment tasks later, and the others finished two duration judgment tasks ahead and two length judgment tasks later.

The tasks varied in relevant dimensions for discrimination (line length or duration), and also varied in the range of exposure durations. There were therefore four conditions that were conducted in separate blocks: *Duration 400–800 ms, Line Length 400–800 ms, Duration 1000–2000 ms, Line Length 1000–2000 ms*. Half of the participants completed two line length judgment tasks ahead and two duration judgment tasks later, and the other half completed two duration judgment tasks ahead and two length judgment tasks later (Figure 4). The order of the two blocks for each task was counter-balanced. Task difficulty was rated after each two blocks ended, in five levels from one to five (1, easy; 2, a bit easy; 3, neither easy nor difficult; 4, a bit difficult; 5, difficult).

**Figure 5:**
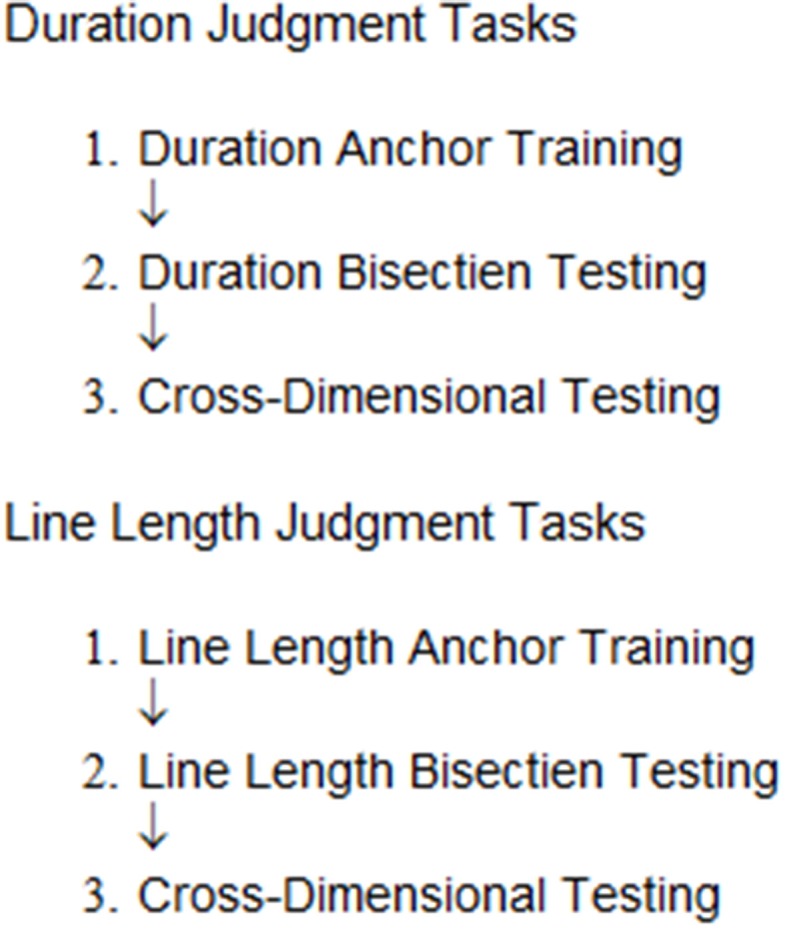
**Procedures of line length and duration judgment tasks**. Each task consisted of following three phases, anchor training, bisection testing and cross-dimensional testing. There were 20 trials in anchor training, and 22 trials in bisection testing and 66 trials in cross-dimensional testing.

At the beginning of the experiments, participants were instructed to keep the same posture and the same position with a constant distance (varied across participants between 30 and 40 cm) from the monitor, to see all stimuli in the same way during tasks. Each task block consisted of three phases that were anchor training, bisection testing and cross-dimensional testing (Figure 5). For duration judgment tasks, the participants were asked not to count, and for duration and line length judgment tasks, they were asked to think anything as much as possible, during the stimulus presentations. The experiments were controlled by a PC with E-prime software (Psychology Software Tools, Inc., USA).

## Duration judgement task

Participants were initially presented with 155 pixel width of line stimuli that were shortest and longest in exposure durations (anchor stimuli; 400 and 800 ms in *Duration 400–800 ms*; 1000 and 2000 ms in *Duration 1000–2000 ms*; see Figure 3) three times each in alternation, and were asked to remember them as the standard for later duration judgments. Before the presentation of the anchor stimuli started, the fixation cross was presented for 1000 ms. The interstimulus interval (ISI) of the anchor stimuli was fixed at 500 ms.

### Anchor training

The participants were trained to judge short or long of exposure durations for the anchor stimuli that was presented once in each trial without any reference stimulus. The stimuli appeared following the fixation cross presented for 1000 ms. Immediately after the disappearance of the stimuli, the participants were asked to respond by pressing one of the two keys (“f” for short and “j” for long).

A visual feedback was given after each response: a red circle for a correct answer and a blue x-mark for a wrong answer. The feedback remained on the screen for 500 ms. In this phase, stimulus value of the irrelevant dimension, the length of line, was fixed at the Middle level (mean; 155 pixel width). There were 20 test trials that were separated into two trial blocks; the short and long anchor stimuli were randomly presented for five times, respectively in one block.

### Bisection testing

The procedure was similar to the anchor training except that the exposure duration of stimuli was varied in seven levels (two anchor and five intermediate levels). The stimuli that have seven different exposure durations were presented in a random order. The number of presentations was differed depending on whether the stimulus was anchor or intermediate levels. In one trial block, the short and long anchor stimuli were presented for three times, and the five intermediate stimuli were presented for once. There were two blocks and 22 trials in total, and participants could take a rest between the blocks. The extent of the irrelevant dimension, line length, was fixed at the Middle level (155 pixel width). The flow of trials was the same as in the anchor training phase. There was a negative/positive feedback only for the anchor stimuli.

### Cross-dimensional testing

The procedure was basically the same as the bisection testing. The exposure duration, the extent of the relevant dimension, was varied in seven levels. The line length, the extent of the irrelevant dimension, was varied in three levels: Short, Middle and Long. In one trial block, each anchor stimulus was presented for three times and each intermediate stimulus was presented once for each level of irrelevant dimension in a random order. There were two blocks of 33 trials in the cross-dimensional testing, and rests were available between blocks. A negative/positive feedback was given only after the anchor stimuli as in bisection testing.

## Line length judgement task

The procedure was basically the same as duration judgment task. The relevant and irrelevant dimensions were interchanged. The “short” and the “long” length of line stimuli (anchor stimuli; 140 pixel and 170 pixel widths; see Figure 3) were initially presented three times each in alternation, and participants were asked to remember them as the standard for later line length judgments.

### Anchor training

The participants were trained to judge short or long of the width for anchor stimuli. A visual feedback was given after each response. In this phase, stimulus values of the irrelevant dimension, the exposure durations, were fixed at the Middle levels (mean; 600 ms in *Duration 400–800 ms*; 1500 ms in *Duration 1000–2000 ms*).

### Bisection testing

The participants judged the width of stimuli varied in seven levels (two anchor and five intermediate levels). The seven different width of line stimuli were presented in a random order. In one trial block, the short and long anchor stimuli were presented for three times, and the five intermediate stimuli were presented for once. There were two blocks and 22 trials in total. The extents of the irrelevant dimension, the exposure durations, were fixed at the Middle levels (mean; 600 ms in *Duration 400–800 ms*; 1500 ms in *Duration 1000–2000 ms*). There was a negative/positive feedback only for the anchor stimuli.

### Cross-dimensional testing

The line length, the extent of the relevant dimension, was varied in seven levels (two anchor and five intermediate levels). The exposure duration, the extent of the irrelevant dimension, was varied in three levels: Short, Middle, and Long. In one trial block, each anchor stimulus was presented three times and each intermediate stimulus was presented once for each level of irrelevant dimension in a random order. There were two blocks of 33 trials in the cross-dimensional testing, and rests were available between blocks. A negative/positive feedback was given only after the anchor stimuli as in bisection testing.

## Results

Before the later analyses, the data of responses that took longer than 4000 ms were excluded as outliers. In trials which reaction time was longer than 4000 ms, the participants might not have concentrated on the stimuli or the task. The data of participants, who did not reach the criteria to judge the anchor stimuli correctly more than 80% in the last 10 trials of anchor training session, were also excluded. Two participants were excluded in *Duration 400–800 ms*, and one subject was excluded in each of the other three tasks.

To assess how saliency of irrelevant stimulus extents would affect on the discrimination of relevant stimuli, the results of combined two tasks *(Duration 400–800 ms* and *Line Length 400–800 ms, Duration 1000–2000 ms* and *Line Length 1000–2000 ms)* in which the rages of stimulus extents were the same for both space and time, were separately analyzed. The 50% points of subjective equality (PSE) were estimated by the maximum likelihood method (Probit Analysis, Finney, 1971; Lieberman, 1983), in all conditions.

### *Duration 400–800 ms* and *Line Length 400–800 ms*

#### Bisection testing

The PSE in *Duration 400–800 ms* was 610.85 ms, and the PSE in *Line Length 400–800 ms* was 154.64 pixels. Reaction time was different between *Duration 400–800 ms* and *Line Length 400–800 ms*; the response in duration judgments took significantly longer than line length judgments [*t*_(20)_ = 4.77, *p* = 0.000] (see **Figure 7**).

#### Cross-dimensional testing

The PSE values were 608.44 ms, 593.85 ms, and 587.86 ms in *Duration 400–800 ms*; 154.48 pixels, 153.81 pixels, and 153.74 pixels in *Line Length 400–800 ms*, for Short, Middle, and Long extents of task irrelevant stimuli. The long line length stimuli were judged longer in duration, and the long exposure duration stimuli were judged longer in line length.

**Figure 6:**
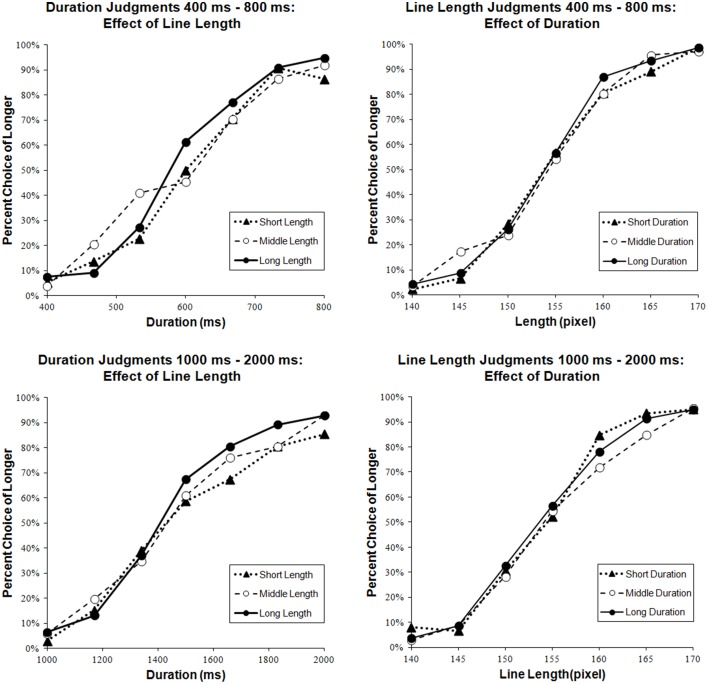
**Effects of irrelevant dimensions**. The graphs on the left side present the data of duration judgment tasks and the graphs on the right side present the data of line length judgment tasks. The upper graphs show the data of tasks whose exposure duration ranged from 400 ms to 800 ms. The lower graphs show the data of tasks whose exposure duration ranged from 1000 ms to 2000 ms. Black triangles (▲), white circles (○), and black circles (●) indicate extents of irrelevant stimuli, Short, Middle, and Long, respectively.

**Figure 7:**
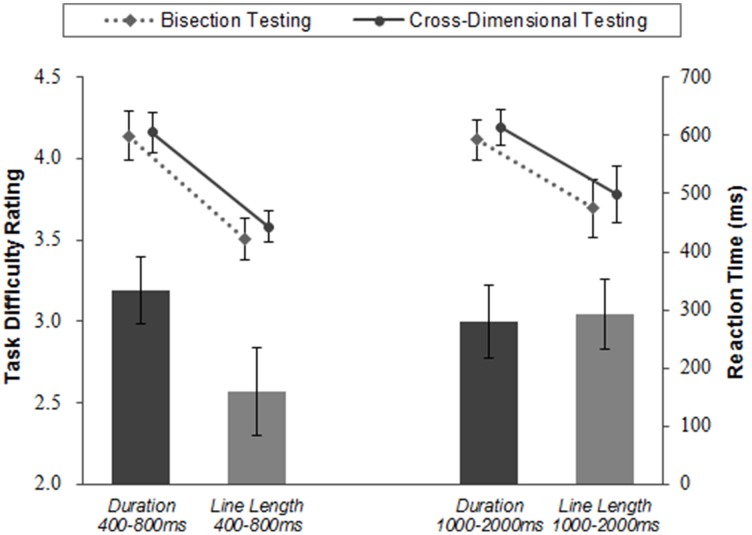
**Task difficulty rating and reaction time**. The left two bars show the task difficulty ratings of duration and line length judgment tasks whose exposure duration ranged from 400 to 800 ms. The right two bars show the task difficulty ratings of duration and line length judgment tasks whose exposure duration ranged from 1000 to 2000 ms. The black bars show the ratings of duration judgment tasks and the gray bars show the ratings of line length judgment tasks. Task difficulty was rated in five levels, from one to five. Line graphs show reaction time in bisection testing and in cross-dimensional testing. Error bars are S.E.M. across participants.

To assess cognitive interactions as effects from the extent of irrelevant dimension on judgments of relevant dimension, a mixed-effects logistic regression was conducted. In the analysis, seven values of the relevant dimension and three values of irrelevant dimension were used as predictors of “short” or “long” responses, and participants were considered as random effects. As a result, the main effect of the irrelevant dimension was significant for *Duration 400-800 ms* [χ^2^_(1, *N*=1451)_ = 4.54, *p* = 0.000] but not for *Line Length 400–800 ms* [χ^2^_(1, *N*=1513)_ = 0.59, *p* = 0.44] (see Figure 6). Reaction time was significantly longer in *Line Length 400–800 ms* than in *Duration 400–800 ms* and *Line Length 400–800 ms* [*t*_(20)_ = 5.73, *p* = 0.000] (see Figure 7).

#### Task difficulty

The averaged task difficulty ratings for *Duration 400–800 ms* was the highest in all conditions, and significantly higher than that of *Line Length 400–800 ms*, according to t-test [*t*_(20)_ = 2.28, *p* = 0.03] (see Figure 7).

### *Duration 1000–2000 ms* and *Line Length 1000–2000 ms*

#### Bisection testing

The PSE for *Duration 1000–2000 ms* was 1530.17 ms, and the PSE for *Line Length 1000–2000 ms* was 153.79 pixels. The response in *Duration 1000–2000 ms* took significantly longer than *Line Length 1000–2000 ms* [*t*_(21)_ = 2.84, *p* = 0.01] (see Figure 7).

#### Cross-dimensional testing

The PSE values were 1507.06 ms, 1466.24 ms, and 1441.01 ms in *Duration 1000–2000 ms*; 153.96 pixels, 155.27 pixels, and 154.25 pixels in *Line Length 1000–2000 ms*, for Short, Middle, and Long extents of task irrelevant stimuli. The long line length stimuli were judged longer in duration, but such tendency could not be observed in line length; the long exposure duration stimuli were not always judged longer.

To assess cognitive interactions, a mixed-effects logistic regression was conducted in the same way as for *Duration 400–800 ms* and *Line Length 400–800 ms*. The main effects of the irrelevant dimension were not significant for *Duration 1000–2000 ms* [χ^2^_(1, *N* = 1501)_ = 0.98, *p* = 0.32] and for *Line Length 1000–2000 ms* [χ^2^_(1, *N*=1505)_ = 0.43, *p* = 0.51] (see Figure 6). The response in *Duration 1000–2000 ms* took significantly longer than in *Line Length 1000–2000 ms* [*t*_(21)_ = 2.52, *p* = 0.02] (see Figure 7].

#### Task difficulty

There was no significant difference between difficulty ratings of *Duration 1000–2000 ms* and *Line Length 1000–2000 ms*, according to *t*-test [*t*_(21)_ = −0.18, *p* = 0.86; Figure 7]. The task difficulties were relatively high in both conditions.

## Discussion

The results of this study supported the hypothesis: asymmetrical space-time interaction is supposed to be caused by the imbalance in saliency of the spatial and temporal information, and difficulties in the spatial and temporal tasks, given the different pattern of results of combined two tasks (*Duration/Line Length 400–800 ms*, and *Duration/Line Length 1000–2000 ms*) in task difficulties and effects from the irrelevant dimension on relevant dimension.

According to the results of a mixed-effects logistic regression, the effect of the irrelevant dimension was the largest in *Duration 400–800 ms* that was the most difficult, and the rating was significantly higher than that of *Line Length 400–800 ms*. On the other hand, the difficulties of *Duration 1000–2000 ms* and *Line Length 1000–2000 ms* were similar. In this case, the effect of the irrelevant dimension on the judgment was not observed. These results can be interpreted as that the balance of difficulty between spatial-temporal cognitive tasks would affect the balance of cognitive interaction.

In discrimination tasks of this study, as already mentioned, when the task difficulty is high, the saliency of relevant stimuli would be low, and vice versa. In the sets of paired spatial and temporal tasks (*Duration/Line Length 400–800 ms, Duration/Line Length 1000–2000 ms*), the ranges of stimulus values were common, therefore the saliency of irrelevant stimuli would be high, when the difficulty of paired task is easy, and vice versa.

In *Duration 400–800 ms*, the task difficulty was high but the difficulty of paired task, *Line Length 400–800 ms* was low, thus the saliency of relevant stimuli was low but the saliency of irrelevant stimuli was high, so that the effect of irrelevant stimuli on the discrimination was statistically significant. In other tasks, the saliency of irrelevant stimuli would not be high enough to affect on the discrimination significantly.

There was a significant difference in reaction time between the spatial and the temporal cognitive tasks. The reaction time of duration judgment was significantly longer than that of line length judgment. This difference in reaction time might reflect the imbalance of stimulus saliency between visual space-time cognitions, as discussed above for the asymmetrical interactions. The processes and/or representations of spatial and temporal information might be partially common or similar (ATOM: Walsh, 2003), although fundamental differences might exist between spatial and temporal cognitions with vision. Such differences may be reflected in reaction time; reaction time of temporal cognitive tasks was longer than that of spatial cognitive tasks, even in the bisection testing in which the irrelevant stimulus extent was fixed at the Middle level, and even though when the task difficulties were similar. The saliency of visual spatial information (the line length extents) would tend to be higher than the saliency of visual temporal information, therefore the automaticity of line length discrimination would tend to be higher than that of duration discrimination via visual perception.

Human adults have well-developed visual perception, and vision dominates over other modalities such as audition, in many cases, in the process to integrate information from several modalities especially in spatial cognition, such as the ventriloquism effect (Thurlow and Jack, 1973). However, in time perception, visual information can be affected by auditory information. It can be seen in temporal ventriloquism and visual illusions by audition, the phenomenon that the number or timing of flashes can be differently perceived from actual vision, which is caused by hearing sounds (Shimojo et al., 2001; Vroomen et al., 2004). In human adults, spatial information via vision tends to be more precise compared to that via audition, and thus vision has predominance over audition in cross-modal spatial cognition. In contrast, the saliency of visual temporal information is low so that audition dominates over vision in cross-modal temporal cognition, in many cases.

In integration of cross-modal information, information with higher saliency would have the predominance over that with lower saliency. As well as in cross-modal perception, in space-time interaction, spatial information affects time information more than vise versa, due to the balance of saliency, in vision of human adults. Such a common hypothesis on integration in cross-modal and cross-dimensional cognitions is supposed to be plausible in terms of ecological validity. Such biases in integration would make the information integration more efficient. In addition, this hypothesis can approach a remaining question: what is the difference between human adults and monkeys? In monkeys, saliency of spatial information from vision might not be so high, or saliency of temporal information from vision might not be so low compared to human adults, or both. Therefore, spatial and temporal information in vision would be reliable to the same degree, which may have led to the symmetrical interaction between spatial and temporal cognition in vision of monkeys, as found in Merritt et al. (2010).

It remains unknown whether it is possible to make the balance between spatial-temporal cognitive interactions in vision reversed: the interaction from time to space is larger than vice versa. Cai and Connell (2015) have proved that the balance of space-time interaction in multi-modal perception can be reversed. So such a reversal in space-time interaction in vision could happen, if the saliency of temporal information becomes higher than that of spatial information. It is still open to the future studies to elaborate the way to assess saliency. In the present study, the task difficulty rating was considered as a related variable to the saliency of judged stimuli, but there could be other ways. It would be better to consider reaction time and other factors such as discrimination sensitivity comprehensively, as well as task difficulty.

### Conflict of interest statement

The authors declare that the research was conducted in the absence of any commercial or financial relationships that could be construed as a potential conflict of interest.
